# Sunlight, dietary habits, genetic polymorphisms and vitamin D deficiency in urban and rural infants of Bangladesh

**DOI:** 10.1038/s41598-022-07661-y

**Published:** 2022-03-07

**Authors:** Subhasish Das, Md. Mehedi Hasan, Minhazul Mohsin, Didarul Haque Jeorge, Md. Golam Rasul, Ar-Rafi Khan, Md Amran Gazi, Tahmeed Ahmed

**Affiliations:** 1 Nutrition and Clinical Services Division, International Centre for Diarrhoeal Disease Research, Bangladesh (icddr,b), Dhaka, Bangladesh; 2 Liggins Institute, University of Auckland, Auckland, New Zealand; 3 Program in Public Health, University of California, Irvine, USA; 4 Department of Global Health, University of Washington, Seattle, WA USA; 5 Department of Public Health Nutrition, James P. Grant School of Public Health, BRAC University, Dhaka, Bangladesh; 6 Office of Executive Director, International Centre for Diarrhoeal Disease Research, Bangladesh (icddr,b), Dhaka, Bangladesh

**Keywords:** Nutrition, Mutation

## Abstract

We conducted an observational study to assess the prevalence and risk factors of vitamin D deficiency in 12–24 months old children living in urban and rural Bangladesh. Serum 25-hydroxyvitamin D (free 25(OH)D) level, socio-demographic status, anthropometric status, dietary intake, exposure to sunlight and single nucleotide polymorphisms in vitamin-D pathway genes were measured in 208 children. Vitamin D deficiency (free 25(OH)D < 50 nmol/l) was reported in 47% of the children. Multivariable logistic regression model identified duration to sunlight exposure (regression coefficient, β =  − 0.01; 95% CI 0.00, − 0.02; p-value < 0.05), UV index (β =  − 0.36; 95% CI 0.00, − 0.02; p-value < 0.05) and breast-feeding (β =  − 1.15; 95% CI − 0.43, − 1.86; p-value < 0.05) to be negatively associated with vitamin D deficiency. We measured the role of single nucleotide polymorphisms in pathway genes (*GC*-rs7041 T > G, rs4588 C > A, *CYP2R1*-rs206793 A > G, *CYP27B1*-rs10877012 A > C and *DHCR7*-rs12785878 G > T) and found statistically significant differences in serum vitamin D levels between various genotypes. SNPs for *CYP27B1* (CA & CC genotype) had statistically significant positive association (β = 1.61; 95% CI 2.79, 0.42; p-value < 0.05) and TT genotype of *GC*-rs7041 had negative association (β =  − 1.33; 95% CI − 0.02, − 2.64; p-value < 0.05) with vitamin-D deficiency in the surveyed children.

## Introduction

Vitamin D plays crucial roles in protecting human body from infection, inflammation and neoplastic diseases^1^. Directly or indirectly, 1, 25-dihydroxy vitamin D modulates the action of more than 200 genes, including genes responsible for the regulation of cellular proliferation, differentiation, apoptosis, and angiogenesis^2–4^. Vitamin D is a well-known regulator of bone mineral metabolism and it also maintains extracellular calcium level^5,6^.

Vitamin D deficiency has emerged as a pandemic among all ages^7^. Globally 1 billion people are suffering from vitamin D deficiency or insufficiency^1^. A study estimated that deficiency in vitamin D is responsible for 4 billion cases of bone diseases and loss of 3·3 billion disability adjusted life years^1^. Vitamin D deficiency might even be found in the sunniest areas of the world if most of the skin remains shielded from the sun due to clothing or indoor activities^8–10^. South Asian countries are no exceptions as a high prevalence of vitamin D deficiency has recently been documented among women and young infants residing within this geography^11^. The prevalence of vitamin D deficiency and insufficiency among pre-school children in Dhaka, Bangladesh varies from 2 to 84%^1^. The Bangladesh National Micronutrient Survey’ 2011–2012 indicated that 32.1% of all the preschool children and 39% of school-age children were living with vitamin D insufficiency^12^. Another study revealed that the prevalance of vitamin D deficiency among underweight children was 40%, which was higher than the prevalence in normal population^13^.

While most of the vitamin D is synthesized by the skin upon exposure to sunlight (UV-B radiation) and photolytic cleavage of 7-dehydrocholesterol to pre-vitamin D3, some fraction of it also comes from the diet^1^. The biologically active form of vitamin D [1α,25-(OH)2 D3] is produced from pro-vitamin D3 through two enzymatic activation steps. *CYP27A1* and *CYP2R1* genes encode 25-hydroxylase that catalyzes the steps required for C-25 hydroxylation of vitamin D3. Then, a final activation enzyme, encoded by *CYP27B1* gene, catalyzes the rate-limiting C-1 hydroxylation step to produce the active form of vitamin D- 1α,25-(OH)2 D3. Vitamin D is then either transported to the target cells by vitamin D binding protein (also known as VDBP/GC) for exerting the desired biological action or is metabolically inactivated by the 24-hydroxylase enzyme (encoded by the *CYP24A1* gene)^14–17^. VDBP is the major transport protein of 25(OH)D3 in the circulation. It regulates the delivery of vitamin D to target cells and thus plays an essential role in maintaining the availability of the micronutrient^18,19^.

As several genes control the pathways of synthesize and transport of vitamin D, alteration in expression of those genes might play crucial role in vitamin D status, in individual and even in population level. But the role of such alterations in development of vitamin D deficiency in Bangladeshi infants, especially in relation to regular exposure to sunlight and dietary intake of vitamin D, has not been reported yet. Moreover, there is a paucity of data regarding the proportionate contribution of sunlight exposure and dietary intake on vitamin D deficiency among Bangladeshi children. To fill this gap we did an observational study to measure the association of sunlight exposure, dietary intake and expression of vitamin D pathway genes in serum vitamin D deficiency of urban and rural children living in Bangladesh.

## Methods

### Study settings and participants

We conducted a survey among 12 to 24 months old children of Bauniabadh and adjacent slum area in Mirpur, Dhaka (urban site) and Matlab, Chandpur (rural site) of Bangladesh. A study done in Bauniabadh, Mirpur found a 40% prevalence of vitamin D deficiency among 6–24 month old children^1^. Using this proportion as the assumed prevalence of vitamin D deficiency and using the formula for cross sectional study design (n = Z_1−α/2_^2^ P (1 − P)/d^2^, where Z_1−a/2_ = 1.96, precision, d = 0.1, and proportion, P = 0.4), our required sample size was, n = (1.96)^2^ × 0.4 × (1 − 0.4)/(0.1)^2^ = 93. Adding an attrition of 10%, the final sample size was 104 for each site. Hence, we considered recruiting a total of 208 (minimum) children for this study. Children with history of vitamin D deficiency, rickets, vitamin D or calcium supplementation, any congenital malformation, history of seizure, chronic disorders, presence of severe acute malnutrition (mid-upper arm circumference < 11.5 cm or Weight-for-Height Z-Score < ** − **3), skin disorder such as atopic dermatitis and presence of severe anaemia were excluded from the sampling list. Based on the above-mentioned criteria the field research assistants screened and recruited the children into the study. Written informed consents were taken from the parents/caregivers prior to enrolling the child. The study was approved by the Institutional Review Board, International Centre for Diarrhoeal Disease Research, Bangladesh (icddr,b) (protocol number PR-18069) and all methods were carried out in accordance with relevant guidelines and regulations.

### Data collection

#### Dietary intake data

24-h dietary recall and food frequency questionnaires were used for documenting dietary intake of vitamin D. Trained field workers used the 24-h multiple-pass dietary recall approach to collect a variety of detailed information about the foods consumed over last 24 h^20^. Dietary intake data was then converted to nutrients using a locally adapted food composition table and thus the dietary intake of vitamin D was calculated. Frequency of consumption of vitamin D-rich foods during the last 24-h was documented using a pre-tested food frequency questionnaire (adopted from Demographic and Health Survey questionnaires)^21^.

#### Daily sunlight exposure and UV index data

Solar zenith angle has a substantial impact on ultraviolet B (UVB) radiation. During early morning and late afternoon the solar zenith angles remain oblique and the number of UVB photons that reaches the earth’s surface decreases^22,23^. As a result a lower amount of vitamin D is produced during this time^24^. It is evident that between 11 a.m. and 2 p.m. maximum amount of sunlight reaches to South Asian regions and exposure to sunlight between the hours of 11 a.m. and 2 p.m. is expected to promote maximum vitamin D production in the skin^25,26^. Hence, we collected daily sun exposure data from each child between 11 am and 2 pm on 3 different days in 3 consecutive weeks. During each data collection sessions trained study personnel observed the child without notifying the mother and marked the child’s body surface area exposed to sunlight using the Lund and Browder chart^27^. The data collectors then interviewed the mother/caregiver of the participants daily to record the amount of time (in minutes) the child remained under the sun. But, a study also showed significant seasonal variation in solar irradiation in Bangladesh^28^. Based on the comparison of different estimated and measured global solar radiation, the study found that months in Bangladesh could be categorized into three groups—group 1: March–May, group 2: October–January and group 3: February, June–September. Keeping that in mind, to minimize the effect of seasonality due to variation in solar radiation, data collection year was divided into three seasons, season 1 (March–May), season 2 (February, June–September), and season 3 (October–January). Site and date specific UV index data was collected from Weather Online Meteorological Services Ltd (https://www.weatheronline.co.uk/), a meteorological data provider based in UK. The data was then merged with the main data set according to the dates when daily sunlight exposure data was collected from a child. The participant-specific mean values of daily sun exposure and UV index data that was collected over three seasons was used for the final analysis.

#### Serum vitamin D status

After collecting the above-mentioned observational data, blood samples were collected from each child for assessing the serum vitamin D [25(OH)D] status and for analysing the status of genetic polymorphism. A total of 5 ml venous blood was collected aseptically from each child and 4 ml was transferred to collection vials for total nucleic acid isolation. The remaining amount (1 ml) was centrifuged for the separation of plasma and subsequent measurement of vitamin D using a commercial ELISA kit (ALPCO, CAT# 38-KAPF1991). The free 25(OH) vitamin D ELISA is a quantitative immunoassay for in vitro determination of the concentration of free 25(OH) vitamin D in human serum.

#### DNA collection, SNP selection, and genotyping

Genomic DNA was extracted from buffy coat using QIAamp DNA Blood mini-Kit (Valencia, CA, USA) as per manufacturer’s protocols. DNA was then quantified using microplate Spectrophotometer (NanoDrop; Thermo Fisher Scientific Inc.) with a requirement that the A260/A280 ratio was in the range of 1.8–2.1. DNA stock aliquots were diluted to a concentration of 50 ng/µl and were frozen until needed for PCR assays. We selected four candidate genes containing 5 SNPs according to the following criteria: (1) biological importance in vitamin D metabolism and transportation (2) evidence of a significant association to vitamin D deficiency according to previous GWAS reports and (3) having significant relation with vitamin D deficiency in the population who were genetically close and environmentally similar to Bangladeshi population^14,29–31^. The selected genes and SNPs were *GC* (rs7041T > G, rs4588C > A), *CYP2R1* (rs206793A > G), *CYP27B1* (rs10877012 A > C) and *DHCR7* (rs12785878G > T). The list of the genes with SNPs and genotypes (wild type, heterozygous mutant and homozygous mutant) are presented in Table 1 of supplementary file 1. The PCR technique was used for genotyping followed by restriction fragment length polymorphism assays (PCR–RFLP). The PCR-specific primer sets, and restriction enzymes were selected based on the evidence of previous studies and are showed in Table 2 of supplementary file 1^14,31–33^. The digestion products were electrophoresed on 2% agarose gels that was stained with ethidium bromide and later the plate was visualized under UV light.

#### Anthropometry, socio-economic status, serum haemoglobin level

Two trained field research assistants followed standard operating procedures to measure the anthropometric indices (length, weight, head circumference, mid-upper arm circumference and triceps skinfold thickness) of the children on the day of enrolment^34^. Every measurement was taken twice, and the mean value was documented. Socio-economic status data was collected using a questionnaire that was adopted from Demographic and Health Survey questionnaires. Capillary blood was collected and measured in Hemocue device (Hb 201, Ängelholm, Sweden) for estimation of haemoglobin level. We did not collect skin colour tone data as no or very little variation was expected in skin colour of the participants as they were from same ethnic backgrounds. The skin tone of this ethnic group falls under Fitzpatrick type V^35,36^.

### Data management and statistical analyses

Serum vitamin D deficiency, the primary outcome variable for this study was defined as having a serum vitamin D level < 50 nmol/l. To summarize the data, proportion estimates was used for categorical variables, mean estimates with standard deviations was used for symmetric quantitative variables and median values were reported for asymmetric quantitative variables. t-test/Mann–Whitney/Kruskal–Wallis test was applied for group wise comparison of quantitative variables and chi-square test was used for categorical variables. A conceptual framework that was developed (supplementary file 2) based on the biological pathway of vitamin-D metabolism was used for variable selection. Bivariate and multivariable logistic regression models were fit for the binary outcome variable (vitamin D deficient yes vs. no) to identify the factors associated with serum vitamin D deficiency. In the logistic regression models, regression coefficients with 95% CIs were generated and reported. A case–control approach was taken for measuring the association of single nucleotide polymorphisms in pathway genes to vitamin D deficiency, and top 25% children (based on their serum vitamin D levels) were compared to their bottom 25% counterparts where the wild types of the genes were counted as the reference categories. A probability value (p-value) < 0.05 was selected as a cut-off for statistical significance for all the analyses. R statistical software version 4.0.5 was used for data management and analysis.

## Results

The socio-demographic and anthropometric characteristics of the study participants are presented in Table 1. Most of the children (75.5%) were from male-headed families, and more than 90% of the families reported a monthly income of > 100 USD. Maternal age was between 18 and 30 years during birth of the enrolled child for more than three-fourth of the participants. Mean length-for-age z-score was − 1.32 (s.d. 1.10), mean weight-for-age z-score was − 0.83 (s.d. 1.14) and mean weight-for-length z-score was − 0.24 (s.d. 1.11). Nearly half (48%) of the study participants were suffering from vitamin D deficiency. The overall mean (SD) level of serum vitamin D level was 51.3 (14.7) nmol/l. The non-deficient group had a vitamin D level of (mean ± s.d.) 62.7 ± 9.55 nmol/l and the deficient group had a mean of 39.1 nmol/l (s.d. 7.71 nmol/l). Prevalence of vitamin D deficiency was higher among the female children (53.1%) compared to the male children (41.75%). However, the prevalence was almost similar among the urban and rural children and between children from male- and female-headed families. Children from the mothers who completed at least primary education suffered less in vitamin D deficiency (43.22%) compared to the children whose mothers were less educated. Half of the children were found to be vitamin D deficient when the childbirth age of their mother was less than 18 years. However, none of these socio-economic and socio-demographic characteristics showed any statistically significant difference between the groups. The trend was similar for the nutritional status indicators too and we observed no statistically significant differences in anthropometric characteristics of the surveyed children based on their vitamin D deficiency status.

**Table 1:** Baseline socio-economic and anthropometric characteristics of the participants.

	All (n = 216)	Not-deficient (n = 113)	Deficient (n = 103)	p-value
	n (%)			
**Sex**				
Male	103	60 (58.25%)	43 (41.75%)	0.10
Female	113	53 (46.9%)	60 (53.1%)	
**Place of residence**				
Urban	117	61 (52.14%)	56 (47.68%)	0.96
Rural	99	52 (52.53%)	47 (47.47%)	
**Household head’s sex**				
Male	163	85 (52.15%)	78 (47.85%)	0.93
Female	53	28 (52.83%)	25 (47.17%)	
**Mother’s age at birth (years)**				
< 18 years	12	6 (50%)	6 (50%)	0.94
18-30 years	167	90 (53.89%)	77 (46.11%)	
> 30 years	33	17 (51.52%)	16 (48.48%)	
**Mother’s age at first pregnancy**				
< 18 years	63	38 (60.32%)	25 (39.68%)	0.18
≥ 18 years	149	75 (50.34%)	74 (49.66%)	
**Maternal education**				
No education	21	11 (52.38%)	10 (47.62%)	0.49
< 5 years	73	35 (47.95%)	38 (52.05%	
> 5 years	118	67 (56.78%)	51 (43.22%)	
**Separate room for a kitchen**				
Yes	202	104 (51.49%)	98 (48.51%)	0.35
No	14	9 (64.29%)	5 (35.71%)	
**Monthly family income**				
< 100 USD	20	11 (55%)	9 (45%)	0.80
> 100 USD	196	102 (52.04%)	94 (47.96%)	
**Treat water to make it safe**				
Yes	112	58 (51.79%)	54 (48.21%)	0.87
No	104	55 (52.88%)	49 (47.12%)	

	Mean (SD)			
Serum vitamin D levels (nmol/l)	51.3 (14.7)	62.7 (9.55)	39.1 (7.71)	< 0.05
Weight (kg)	10.14 ± 1.62	10.02 ± 1.54	10.28 ± 1.70	0.23
Length (cm)	79.70 ± 4.46	79.47 ± 4.33	79.94 ± 4.61	0.44
MUAC (cm)	14.82 ± 1.17	14.77 ± 1.21	14.88 ± 1.14	0.34
Head circumference (cm)	45.50 ± 1.43	45.40 ± 1.39	45.60 ± 1.47	0.30
Triceps skinfold thickness (mm) left arm	7.48 ± 1.42	7.46 ± 1.33	7.50 ± 1.52	0.99
Triceps skinfold thickness (mm) right arm	7.65 ± 1.45	7.63 ± 1.36	7.67 ± 1.56	0.91
Length-for-age Z score	− 1.32 ± 1.10	− 1.38 ± 1.02	− 1.26 ± 1.19	0.44
Weight-for-age Z score	− 0.83 ± 1.14	− 0.92 ± 1.08	− 0.72 ± 1.20	0.24
Weight-for-length Z score	− 0.24 ± 1.11	− 0.33 ± 1.11	− 0.14 ± 1.09	0.25
Triceps skinfold-for-age Z score (right)	7.64 ± 1.45	7.62 ± 1.36	7.67 ± 1.56	0.91
Triceps skinfold-for-age Z score (left)	7.48 ± 1.42	7.46 ± 1.33	7.50 ± 1.52	0.99
Haemoglobin level	10.81 ± 1.30	10.67 ± 1.34	10.96 ± 1.26	0.10

Table 2 presents the consumption status of vitamin D-rich foods in the last 24-h. We found that a significantly (p < 0.01) higher proportion of children (81%) in vitamin D non-deficient group were being breastfed during the survey than their deficient counterparts (65%).

**Table 2:** Consumption of vitamin D-rich foods in the last 24 h by vitamin D status among 12–24-month-old Bangladeshi children.

Food item (response: yes)	All (n = 216)	Deficient (n = 103)	Non-deficient (n = 113)	p-value
	n (%)	n (%)	n (%)	
Currently breast-feeding	159 (76.6)	67 (65)	92 (81.4)	0.01
Powdered or fresh animal milk	86 (39.8)	38 (36.9)	48 (42.5)	0.40
Infant formula	3 (1.4)	1(1)	2 (1.8)	0.90
Organ meat (liver, kidney, heart)	8 (3.7)	4 (3.9)	4 (3.5)	0.90
Any meat (chicken, beef, lamb, goat, duck)	30 (13.9)	10 (9.7)	20 (17.7)	0.09
Eggs	93 (43.1)	45 (43.7)	48 (42.5)	0.86
Fresh or dried fish	88 (40.7)	49 (47.6)	39 (34.5)	0.05
Dairy products (cheese, yoghurt)	7 (3.2)	4 (3.9)	3 (2.7)	0.71

**Figure 1:**
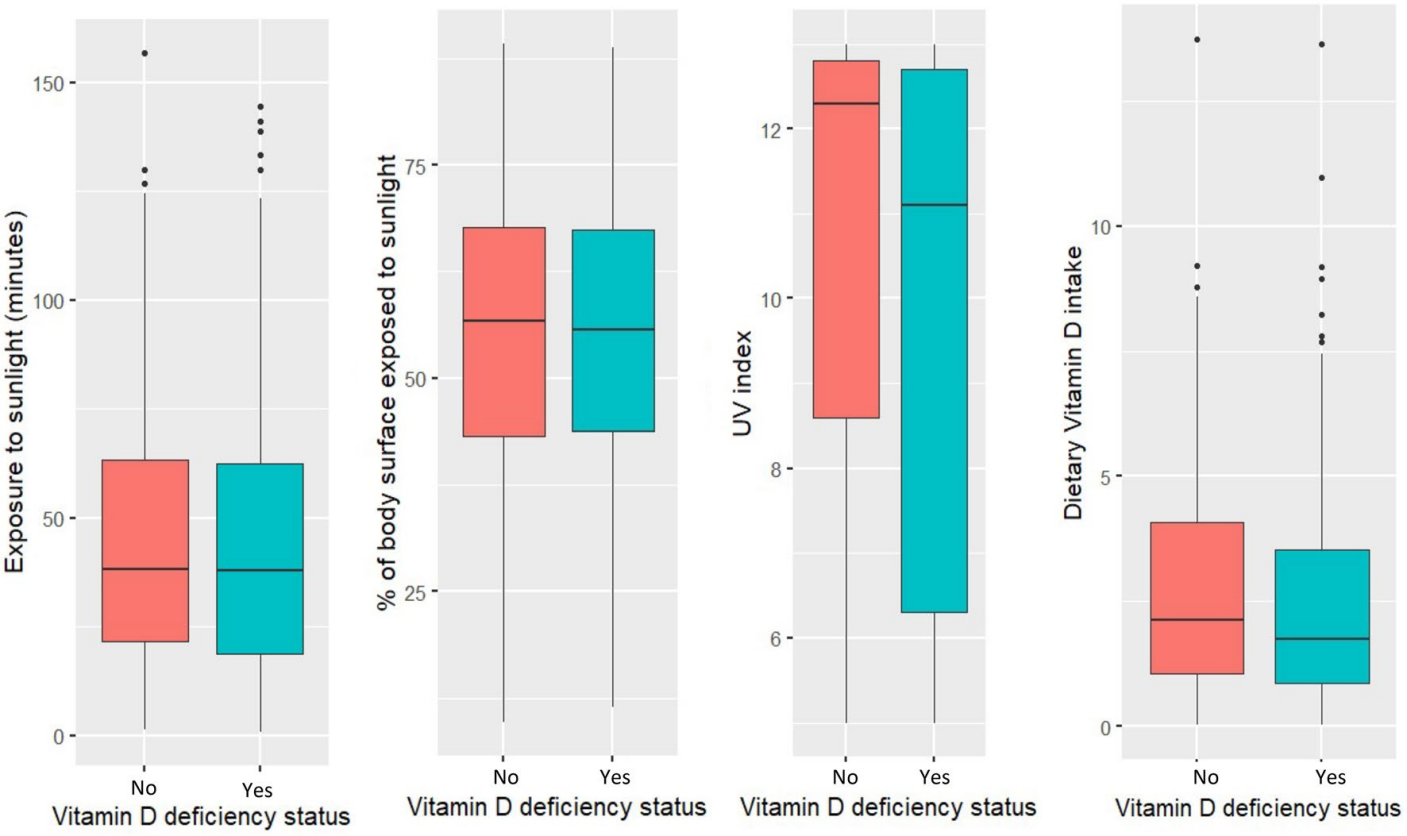
Serum vitamin D levels in relation to exposure to sunlight, percent of body surface exposed to sunlight, UV index, and dietary vitamin D intake.

The boxplots in Fig. 1 show serum vitamin D levels in deficient and non-deficient groups based on exposure to sunlight (in minutes), percent of body surface area exposed to sunlight, UV index, and dietary intake of vitamin D. Though we did not find any significant differences between the groups in any of the variables except UV index (p < 0.01) levels, in all the cases the non-deficient groups had higher median values than their deficient counterparts.

Table 3 presents the results of the multivariable logistic regression model that was fit to identify the factors associated to vitamin D deficiency among the study participants. The results of multivariable regression model reveal that the exposure time (regression coefficient, β =  − 0.01; 95% CI 0.00, − 0.02; p-value = 0.04), UV index (β =  − 0.36; 95% CI 0.00, − 0.02; p-value = 0.02) and current breast-feeding status (β =  − 1.15; 95% CI  − 0.43, − 1.86; p-value = 0.00) had significant negative association to vitamin D deficiency. Although association of exposure score was not statistically significant, it showed a positive trend (β = 0.02; 95% CI 0.04, 0.00; p-value = 0.05). We could not detect any significant association between rural site (β =  − 0.08; 95% CI 0.65, − 0.80; p-value = 0.84), seasonal variety [season 2(β = 0.23; 95% CI = 0.99, − 0.54; p-value = 0.56); season 3 (β = 0.95; 95% CI 0.69, − 2.59; p-value = 0.26), vitamin D intake (β =  − 0.08; 95% CI 0.03, − 0.20; p-value = 0.16) and vitamin d deficiency.

**Table 3:** Factors associated with vitamin D deficiency among 12–24-month-old children living in an urban and rural Bangladesh (Ref. Non-deficient).

	Unadjusted					Adjusted				
	Coefficient	Std. error	95% CI		p-value	Coefficient	Std. error	95% CI		p-value
			Upper	Lower				Upper	Lower	
% of body surface area exposed to sun light	− 0.002	0.01	0.01	− 0.02	0.80	0.02	0.01	0.04	0.00	0.05
Exposure time	− 0.001	0.004	0.01	− 0.01	0.71	− 0.01	0.01	0.00	− 0.02	0.04
Uv index	− 0.11	0.05	− 0.02	− 0.20	0.02	− 0.36	0.15	− 0.06	− 0.65	0.02
Rural site	− 0.02	0.27	0.52	− 0.55	0.96	− 0.08	0.37	0.65	− 0.80	0.84
Season 2	0.12	0.35	0.81	− 0.57	0.73	0.23	0.39	0.99	− 0.54	0.56
Season 3	0.66	0.35	1.34	− 0.03	0.06	− 0.95	0.84	0.69	− 2.59	0.26
Vit D intake	− 0.03	0.05	0.07	− 0.13	0.57	− 0.08	0.06	0.03	− 0.20	0.16
Currently breast feeding	− 0.86	0.32	− 0.23	− 1.48	0.01	− 1.15	0.37	− 0.43	− 1.86	0.00

**Figure 2:**
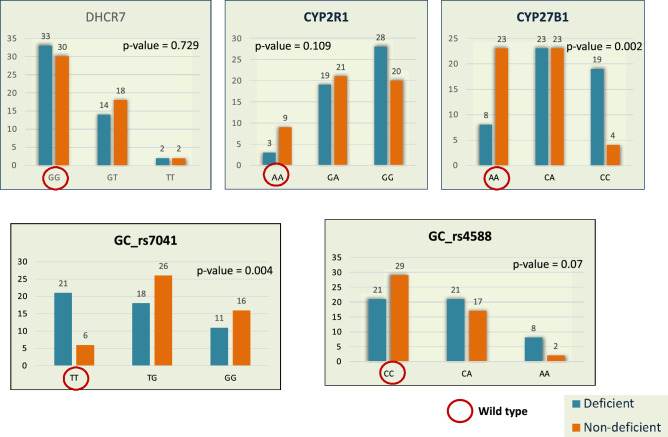
Distribution of different genotypes in deficient (n = 50) and non-deficient (n = 50) groups (p-values were generated from Chi-square test/Fisher exact test).

Figure 2 shows the genotyping distribution of different SNPs in the studied children based on their absolute numbers. Genotypes for *DHCR7* (p-value = 0.729), *CYP2R1* (p-value = 0.109) and *GC*-rs4588 (p-value = 0.07) did not reveal statistically significant differences between the study groups. However, statistically significant differences were seen between the genotypic groups of *CYP27B1* (p-value = 0.0002) and *GC*-rs7041 (p-value = 0.004) genes as more children from the deficient group have CC genotype of *CYP27B1* and TT genotype of *GC*-rs7041 genes.

**Figure 3:**
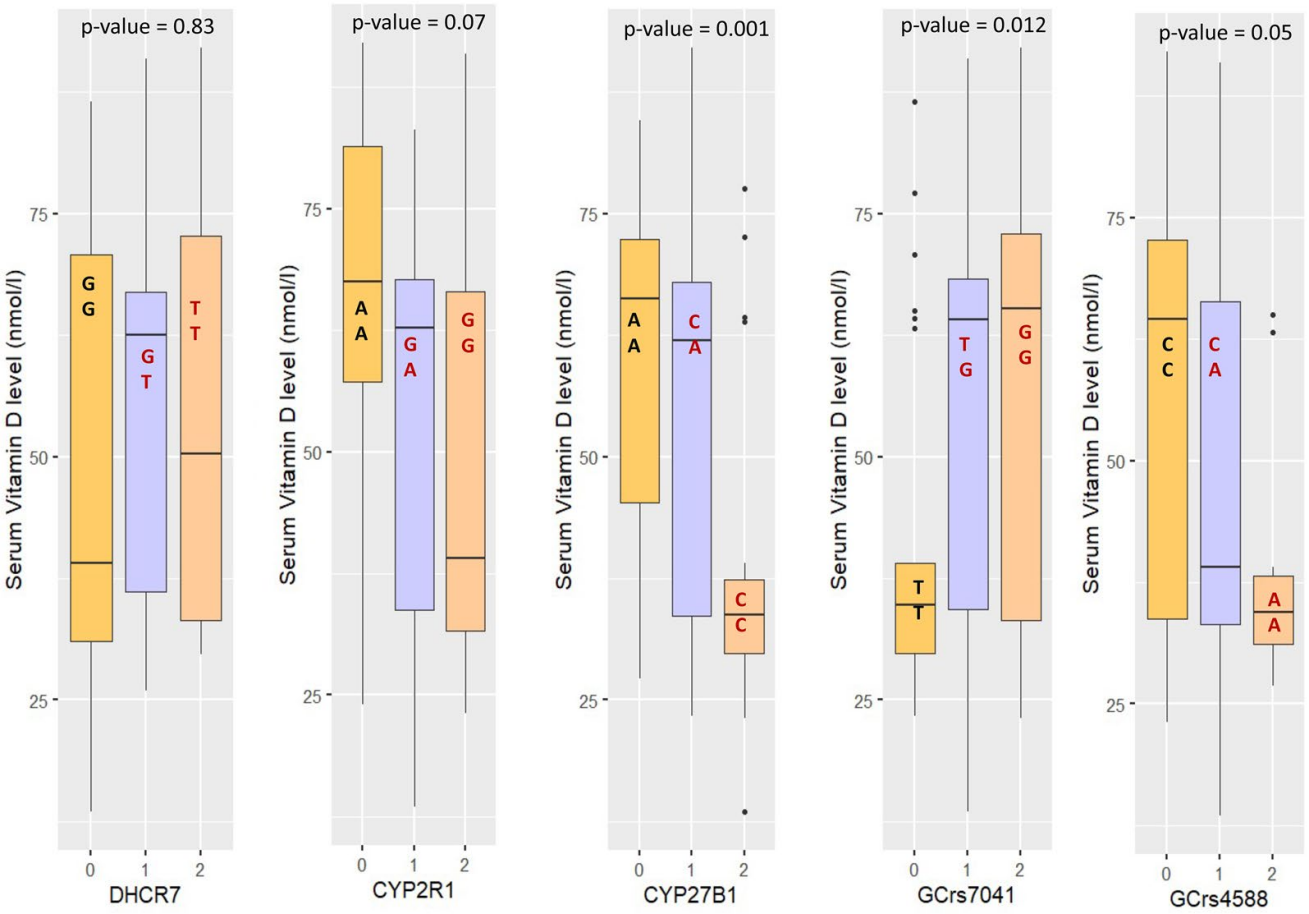
Levels of vitamin-D in the study groups according to the genotypes (p-values were generated from Kruskal–Wallis test).

Figure 3 shows the levels of vitamin-D between the study groups according to their genotypes. Vitamin-D level for TT genotype was comparatively higher (p-value = 0.83) than other genotypes of *DHCR7* gene. Moreover, children having the AA genotype of *CYP2R1* (p-value = 0.07) and *CYP27B1* (p-value = 0.001) gene had higher levels of vitamin D than children who had other genotypes. In addition, vitamin-D level was higher in GG and CC genotypes of *GC*-rs7041 (p-value = 0.012) and *GC*-rs4588 (p-value = 0.05) SNPs, respectively. However, CC genotype of *CYP27B1*, TT genotype of *GC*-rs7041 and AA genotype of *GC*-rs4588 were found to be responsible for the lower level of Vitamin-D in the study participants.

Table 4 shows the association of different SNPs to vitamin-D deficiency. SNPs for *DHCR7*, *CYP2R1* and *GC*-rs4588 genes were not statistically significantly associated to vitamin-D deficiency. However, SNPs for *CYP27B1* and *GC*-rs7041 were found to be significantly associated with vitamin-D deficiency where SNP for *CYP27B1* (β = 1.61; 95% CI 2.79, 0.42; p-value < 0.05) was positively and *GC*-rs7041 (β =  − 1.33; 95% CI  − 0.02, − 2.64; p-value < 0.05) was negatively associated with it.

**Table 4:** Association of SNPs to vitamin D deficiency among 12–24-month-old children living in an urban and rural Bangladesh (Ref. Wild type).

	Unadjusted					Adjusted				
	Coefficient	Std. error	95% CI		p-value	Coefficient	Std. error	95% CI		p-value
			Upper	Lower				Upper	Lower	
*DHCR7* (SNP-Present)	− 0.33	0.43	0.51	− 1.18	0.436	− 0.64	0.53	0.41	− 1.69	0.231
*CYP2R1* (SNP-Present)	1.22	0.70	2.60	− 0.16	0.082	1.00	0.90	2.76	− 0.76	0.265
*CYP27B1* (SNP-Present)	1.42	0.48	2.37	0.47	0.003	1.61	0.61	2.79	0.42	0.008
*GC*-rs7041 (SNP-Present)	− 1.65	0.53	− 0.61	− 2.68	0.002	− 1.33	0.67	− 0.02	− 2.64	0.046
*GC*-rs4588 (SNP-Present)	0.81	0.42	1.63	− 0.01	0.053	0.28	0.59	1.43	− 0.86	0.63

## Discussion

Our study result demonstrated that 47% of the children were suffering from vitamin D deficiency. The multivariable regression model revealed that exposure time to sunlight (p-value = 0.04), UV index (p-value = 0.02) and breastfeeding (p-value = 0.00) were negatively associated to vitamin D deficiency while dietary intake status was not associated with the outcome. We also found that GG allele of *GC*-rs7041 (p-value < 0.05) was negatively and the CA & CC alleles of *CYP27B1*-rs10877012 (p-value < 0.05) were positively associated with vitamin D deficiency in the surveyed children. To our knowledge, this is the first study that has explored the association of sunlight, diet, and genetic polymorphisms to vitamin D deficiency in Bangladeshi children.

Lack of sun exposure is the primary cause of epidemic low vitamin D status worldwide. But the issue has been overlooked in Bangladesh and in many Asian countries, perhaps on the assumption that vitamin D deficiency is unlikely to occur in regions where plentiful sunshine is available year-round. Bangladesh, due to its geographical location gets ample sunshine for most days of the year. But, similar to other South Asian countries the prevalence of vitamin D deficiency in young Bangladeshi children is also very high^37^. We have found a similar prevalence of vitamin D deficiency among children living in urban and rural areas of Bangladesh which is consistent with a study done in urban Bangladesh where the author found that 45.6% children with normal anthropometric indices were suffering from vitamin D deficiency^1^. Previous reports from other South Asian countries (India and Pakistan) also echo our findings^38–41^.

In this study, we systemically measured the children’s exposure time to sunlight and collected UV index data to assess their relationship with vitamin D deficiency. Cutaneous synthesis through the effect of UVR on 7-dehydrocholesterol is the main source of vitamin D for our body and time exposed under the sun and ultraviolet exposure were two very important variables to be assessed. We found a negative association between exposure time to sunlight and vitamin D deficiency. The finding is scientifically supported by the work done by Holick et al. who reported that determinants for the variation of vitamin D status in South-East Asian population were skin pigmentation, lack of sun exposure, the sun protection behaviours such as application of a sunscreen etc^42^. Specker et al. found that serum 25(OH)D level in infants was significantly related to UV exposure^43^. A study done among the breast-fed children of Delhi, India reported a positive correlation of cumulative sun index (a composite score calculated using overall duration, time and body surface area exposed to sunlight) to infant’s serum 25(OH)D level at 6 months of age^26^. Another study conducted in Sweden showed that the UV dose had a small, but significant positive effect on the vitamin D levels^44^. Experimental studies also found positive effects of sun exposure on immune functions that works through both vitamin D and non-vitamin D dependent pathways^45^.

In this cohort of children, breast feeding was found to be negatively associated to vitamin D deficiency and a significantly lower number of children who were being breast-fed during the survey period suffered from vitamin D deficiency than their non-breast-fed counterparts. A longitudinal, randomized, double-blind, placebo-controlled trial done in Madison, Wisconsin, USA reported significantly higher serum concentrations of 25-hydroxyvitamin D3 in the un-supplemented human milk-fed group compared to the supplemented group^46^. Moreover, a summary of relevant studies done in United States of America, New Zealand, Arabian region and Pakistan revealed positive relations between high prevalence vitamin D deficiency among lactating mothers to the high prevalence of vitamin D deficiency in their breast-fed infants who also lack sun exposure^47^. Human milk contains different metabolites of vitamin D such as unconjugated 25-OHD, 24,25(OH)2D, and 1,25(OH)2D^48^. The biologically plausible findings of our study indicate that vitamin D deficiency among breastfed infants should be rarely found. However, if an infant does not receive additional vitamin D from complementary foods, or adequate exposure to sunlight, or the mother is vitamin D deficient, the child might suffer from vitamin D deficiency. In our study we did not find any significant association between dietary intake of vitamin D and vitamin D deficiency. This might be because all the surveyed children, irrespective of their vitamin D status, took much lower vitamin D than their Recommended Dietary Allowance (RDA). Generally, the food items that are good sources of vitamin D are not many in numbers and hence, even with a balanced diet it might be difficult to ensure recommended amount of vitamin D from regular dietary sources. According to Institute of Medicine guidelines the RDA of vitamin D for 12–24 months old children is 600 IU/day^49^. But, in our study, the mean vitamin D intake was much lower than the RDA of vitamin D. Related scholarly evidences also suggest that typical daily intake of vitamin D from food contributes less than UVB sunlight exposure to average year-round 25(OH)D levels^50^.

In our study we found that children with wild type genotypes of *CYP2R1*-rs2060793 (AA), *CYP27B1*-rs10877012 (AA) and *GC*-rs4588 (CC) had higher levels of vitamin D. In contrast, children with wild type genotypes of *GC*-rs7041 (TT) and *DHCR7*-rs12785878 (GG) had lower vitamin D levels. *CYP2R1* gene which was found to be associated with circulating level of 25(OH) D encodes vitamin-D-25-hydroxilase enzyme^51–53^. In our study, higher level of 25(OH)D was found in children with AA genotype of *CYP2R1* whereas lower level was observed in GG genotype. These findings are in line with the results of another study where a higher level of 25(OH)D was found in the study participants with AA genotype while lower level was observed in GG genotype^54^. *CYP27B1* gene encodes the vitamin-D-1α-hydroxylase which converts 25-(OH) D into active vitamin D metabolite 1,25(OH)_2_ D_._ This gene has been found to be strongly associated with vitamin-D status^55^. Our study found that children with AA genotype of *CYP27B1* gene had higher level of 25(OH) D compared to CC and CA genotypes. These findings are supported by other study where higher level of 25(OH) D was found for AA genotype and lower level for CC genotype in sarcoidosis patient^52^. *GC* gene encodes the vitamin-D binding protein (DBP) that is responsible for binding and transportation of vitamin D and its metabolites^56^. When 25(OH)D binds to DBP, it becomes less susceptible to hydroxylation and degradation. Hence, DBP acts as a 25(OH)D depot and supports stabilization and maintenance of 25(OH)D concentration in body pool^57^. In our study, children with CC genotype of *GC*-rs4588 showed higher 25(OH)-D level, whereas lower level was found in case of AA genotype. These results are consistent with the findings of other two studies where higher level of serum vitamin-D was found in CC genotype and lower level in AA genotype^52,58^. In case of *GC*-rs7041, T allele was found to be associated with lower level of 25(OH)D^59^ that echoes our finding. Another study done in Danish children echoed our findings too as they reported that *GC*-rs4588 and rs7041 were associated with lower concentrations of free serum 25(OH)D across the school year^60^. Reports from two large genome-wide association studies have also found that *GC*-rs2282679, which is the near proxy for rs4588 and rs7041 were most strongly associated with serum 25(OH)D levels^51,61^. We also surveyed the role of *DHCR7* in vitamin D deficiency in children. *DHCR7* modulates the catabolism of cholesterol from 7-DHC by encoding the enzyme 7-dehydrocholesterol reductase and thus removes a key substrate required for the vitamin D synthesis^51^. Studies reported that SNPs for *DHCR7* gene were found to be associated with the level of 25(OH)D in serum^62,63^. In our study, findings regarding *DHCR7* gene were supported by the study conducted by Zhang et al. where they reported that individuals with GG genotype presented lower level of 25(OH)D^62^. On the other hand, TT genotype of *DHCR7* was found to be associated with higher level of 25(OH)D in our study that is opposite of the findings of another study where Cooper et al. reported that T allele carriers had significantly lower level of 25(OH)D in type 1 diabetes patient^63^. Moreover, the multivariable regression model revealed that GG genotype of *GC*-rs7041 and the CA & CC genotypes of *CYP27B1*-rs10877012 were associated with vitamin D deficiency in the surveyed children. In these findings, GG genotype of *GC*-rs7041 was negatively associated with vitamin D deficiency which suggests that GG genotype might play a protective role against vitamin-D deficiency. However, SNP for *CYP27B1* was positively associated with vitamin D deficiency suggesting that these SNPs might be a causative factor for vitamin-D deficiency among the surveyed children.

### Limitations and strength

The study we report here followed a conceptual framework for data collection and analysis that was developed following the biological pathway of vitamin D synthesis. Hence, we were able to explore and adjust for most of the variables that played crucial roles in vitamin D synthesis. But we do have a few limitations to report. Due to the descriptive nature of the study, we could not explore the relationship among the exposure and outcome variables in the context of causality. Our selection and investigation of SNP modification were supported by the results of the observational studies conducted in adjacent geographies. Hence, it is possible that we might have failed to explore the role of other SNPs that might be responsible for vitamin D deficiency among the children we surveyed.

## Conclusion

Our study reports that common variants in vitamin D pathway genes (*CYP27B1* and *GC*) were associated to vitamin D deficiency in rural and urban children of Bangladesh. We also found that breast feeding and duration of sunlight exposure were negatively associated to the outcome. Our findings imply that initiatives need to be taken to increase safe sun exposure by encouraging more outdoor activities. Our result also suggests that focusing more on modification of vitamin D pathway genes might have positive impacts on the vitamin D concentrations. Overall, the findings from this study warrant further longitudinal exploration and genome-wide association studies to reveal the entire spectrum of vitamin D deficiency within the framework of genetic variations across ages and generations.

## Supplementary Information

Supplementary Tables.

Supplementary Figure 1.

## Data Availability

The dataset generated and analysed during the current study is available from the corresponding author on reasonable request.
