# Supplementary material for: Sunlight, dietary habits, genetic polymorphisms and vitamin D deficiency in urban and rural infants of Bangladesh
Source: Sci Rep. 2022 Mar 7;12:3623. doi: 10.1038/s41598-022-07661-y (PMC8901932; doi:10.1038/s41598-022-07661-y)
Supplement: Supplementary file 1 — Supplementary Tables. [file 41598_2022_7661_MOESM1_ESM.docx]

| Supplementary Table 1: Genes with SNPs and genotypes (wild type, heterozygous mutant, and homozygous mutant) | | | | | |
| --- | --- | --- | --- | --- | --- |
| **Genes** | **SNPs** | **Global minor allele frequency (GMAF)*** | **Genotypes** | | |
|  |  |  | **Wild type** | **Heterozygous mutant** | **Homozygous mutant** |
| *DHCR7* | rs12785878 | 0.471 | GG | GT | TT |
| *CYP2R1* | rs2060793 | 0.361 | AA | GA | GG |
| *CYP27B1* | rs10877012 | Not found** | AA | CA | CC |
| *GC* | rs7041 | 0.385 | TT | TG | GG |
| *GC* | rs4588 | 0.216 | CC | CA | AA |

*GMAF for individual SNP was taken from “SNPedia.com”

**We selected *CYP27B1*- rs10877012 (Genotype: AA, CA, CC) from previously published article [[1](#_ENREF_1), [2](#_ENREF_2)]. However, we did not find GMAF for rs10877012 (AA, CA CC genotype) in the “SNPedia.com”.

| Supplementary Table 2: List of PCR-specific primer sets, and restriction enzymes | | | | |
| --- | --- | --- | --- | --- |
| Gene | Region | Primer | Restriction Enzyme | References |
| GC | rs7041  rs4588 | F: AAATAATGAGCAAATGAAAGAAGAC  R: CAATAACAGCAAAGAAATGAGTAGA | Sty I  Hae III | [[3](#_ENREF_3)] |
| CYP2R1 | rs2060793 | F: TTCTAGAGGCTGCCCACATTCCTT  R: GTGTTGCAAAGGCAGGGTTGATCT | Hinf I | [[4](#_ENREF_4)] |
| CYP27B1 | rs10877012 | F: TGA CCT TCA ATT CCA GAA CTT CA  R: GGT GGC GTA TGC CTG TAG TG | Hinf I | [[5](#_ENREF_5)] |
| DHCR7 | rs12785878 | F: CTG TCT TCT CTT AGG AGG TT  R: CAA GCA GCA GAC AGG ACA TGA | TaqI | [[6](#_ENREF_6)] |

**References:**

1. Bailey, R., et al., *Association of the vitamin D metabolism gene CYP27B1 with type 1 diabetes.* Diabetes, 2007. **56**(10): p. 2616-2621.

2. Zhu, Q., et al., *Single-nucleotide polymorphism at CYP27B1-1260, but not VDR Taq I, is possibly associated with persistent hepatitis B virus infection.* Genetic testing and molecular biomarkers, 2012. **16**(9): p. 1115-1121.

3. Li, F., et al., *Vitamin D binding protein variants associate with asthma susceptibility in the Chinese Han population.* BMC medical genetics, 2011. **12**(1): p. 1-7.

4. Haldar, D., et al., *Association of VDBP and CYP2R1 gene polymorphisms with vitamin D status in women with polycystic ovarian syndrome: a north Indian study.* European journal of nutrition, 2018. **57**(2): p. 703-711.

5. Thanapirom, K., et al., *Genetic variation in the vitamin D pathway CYP2R1 gene predicts sustained HBeAg seroconversion in chronic hepatitis B patients treated with pegylated interferon: a multicenter study.* PLoS One, 2017. **12**(3): p. e0173263.

6. Thanapirom, K., et al., *Vitamin D-related gene polymorphism predict treatment response to pegylated interferon-based therapy in Thai chronic hepatitis C patients.* BMC gastroenterology, 2017. **17**(1): p. 1-8.
