# Supplementary material for: Sunlight, dietary habits, genetic polymorphisms and vitamin D deficiency in urban and rural infants of Bangladesh
Source: Sci Rep. 2022 Mar 7;12:3623. doi: 10.1038/s41598-022-07661-y (PMC8901932; doi:10.1038/s41598-022-07661-y)
Supplement: Supplementary file 2 — Supplementary Figure 1. [file 41598_2022_7661_MOESM2_ESM.pdf]

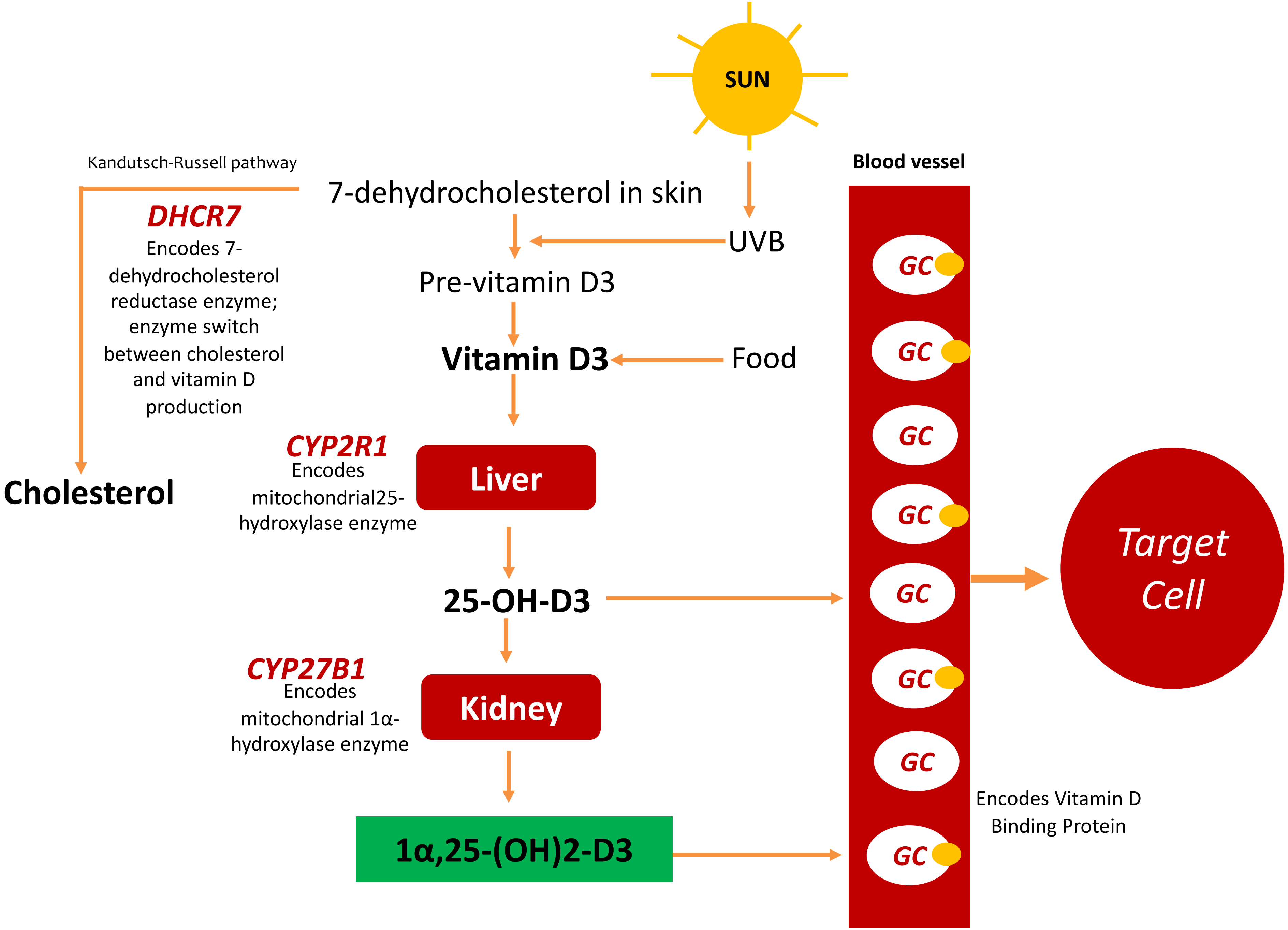

Supplementary figure 1: Conceptual framework presenting the biological pathway of vitamin D synthesis
